# Abdominal Cocoon Syndrome: A Rare Condition That Causes Diagnostic Challenges

**DOI:** 10.5152/tjg.2026.25642

**Published:** 2026-05-04

**Authors:** Zekiye Nur Harput, Orhan Sezgin, Feramuz Demir Apaydın, Enver Reyhan

**Affiliations:** 1 Department of Gastroenterology, Kütahya City Hospital, Kütahya, Türkiye; 2 Department of Gastroenterology, Mersin University School of Medicine, Mersin, Türkiye; 3 Department of Radiology, Mersin University School of Medicine, Mersin, Türkiye; 4 Department of General Surgery, Mersin University School of Medicine, Mersin, Türkiye

To the Editor,

Abdominal cocoon syndrome is a rare and difficult-to-diagnose disease characterized by the complete or partial envelopment of abdominal organs within a membrane.^1^ To increase awareness of this under-recognized condition, we present a patient who was evaluated for years for abdominal pain and experienced recurrent episodes of ileus. He was diagnosed with Behçet’s disease and Crohn’s disease and was even treated using a biological agent. Ultimately, he was diagnosed with an abdominal cocoon, and following definitive surgical treatment, all discomforts of the patient were resolved.

A 27-year-old man presented with a 12-year history of recurrent abdominal pain. He was diagnosed with Behçet’s disease during middle school and was treated with colchicine and steroids until college. His abdominal pain had worsened over the past 6 months. He was hospitalized in the general surgery clinic 4 months earlier for ileus. He improved with symptomatic treatments. During hospitalization in the general surgery department, the patient was unaware of the medications administered and reported that he had not received any analgesic therapy outside this period.

As his symptoms persisted, he was evaluated in the gastroenterology clinic. Physical examination revealed no abnormalities except for abdominal tenderness. Laboratory investigations were completely normal except for a mildly elevated C-reactive protein (CRP) and sedimentation rate (sedimentation rate: 30 mm/h, CRP: 36 mg/dL). The fecal calprotectin level was normal (43 μg/g). Colonoscopy revealed aphthous ulcers in the terminal ileum and linear ulcers in the sigmoid colon. Biopsies from the terminal ileum and sigmoid colon showed only mild inflammation without any significant pathological findings. Tuberculosis was excluded based on negative acid-fast bacilli staining and mycobacterial culture results from terminal ileum biopsies. Oral 5-ASA and azathioprine were initiated due to suspicion of Crohn’s disease. Adalimumab was initiated due to a lack of clinical improvement. His symptoms worsened with treatment. The patient had no history of alcohol use, smoking, or surgery and had discontinued all medications due to a long-standing lack of symptomatic improvement and episodes of voluntary vomiting. He had a family history of rheumatoid arthritis in his sister and ulcerative colitis in his cousin.

The patient presented to our clinic due to persistent symptoms. Abdominal ultrasound revealed mild edema and a clustered appearance of small intestinal loops (Figure 1). Abdominal cocoon syndrome was suspected. The single-balloon enteroscopy was completely normal. Vasculitis and amyloidosis were excluded based on intestinal and colon biopsy results. Computed tomography (CT) enterography revealed findings similar to those detected in ultrasound (Figure 2). Dilatation of the jejunoileal loops, a membranous structure partially encasing the bowel loops and extending toward the mesenteric root, was observed. These findings were suggestive of abdominal cocoon syndrome. Laparotomy was performed. Diffuse adhesions were observed in the abdominal cavity and were carefully lysed. The small bowel loops were found to be encapsulated within a cocoon-like fibrous membrane, which encased the loops and caused luminal narrowing. The small intestine was released from the fibrotic bands. Histopathological examination of the resected tissue revealed fibrosis, hyalinization, and inflammation. The patient was diagnosed with abdominal cocoon syndrome (Figure 3). He experienced no postoperative complications. His abdominal pain resolved completely, and he was able to resume a normal diet. A follow-up colonoscopy performed 6 months after the operation was completely normal. At the 2-year follow-up, the patient remained asymptomatic and in good health. Informed consent was obtained.

Abdominal cocoon syndrome is characterized by the complete or partial enclosure of the abdominal organs within a membrane structure. It was first described by Owtschinnikow^2^ in 1907 with the term peritonitis chronica fibrosa incapsulata.^1^ The term abdominal cocoon was first used by Foo in 1978.^3^-^4^ Although the etiology remains unclear, abdominal cocoon syndrome may be idiopathic or develop due to local or systemic inflammatory processes caused by medications, intra-abdominal infections, mechanical or chemical intraperitoneal irritants, or rheumatological diseases.^1^^,^^5^ ^6^ Some researchers also believe that the disease represents a subtype of retroperitoneal fibrosis.^7^ Abdominal cocoon syndrome has been observed to be on the rise following the coronavirus disease 19 pandemic, which affected the entire world. This is explained by the hypothesis that the virus triggers pro-fibrotic macrophages, leading to effects on the gastrointestinal system similar to those observed in pulmonary fibrosis.^8^ The true prevalence of the disease is not clearly known. However, a review of cases reported in the literature suggests that it is more common in tropical and subtropical countries and has a male predominance.^1^^,^^9^ As the clinical presentation often presents as an acute abdomen caused due to intestinal obstruction, diagnosis can be made with contrast-enhanced CT and confirmed during laparotomy.^10^ As observed in our patient, the presence of the typical band structure encasing the intestines, resulting in a clustered bowel appearance on abdominal ultrasonography and subsequent CT imaging, may strongly suggest the diagnosis. Increased peritoneal contrast enhancement, thickening, and peritoneal calcifications can be observed on CT. The characteristic finding in abdominal cocoon syndrome is focal encapsulation of the intestines. As the disease progresses, a cocoon-like appearance and dense fibrosis develop.^7^ Although the interval between symptom onset and diagnosis may be longer in relatively milder forms, the largest series in the literature, which included 65 patients, reported a mean duration of 3.9 years.^9^

Colonoscopy revealed aphthous ulcers in the terminal ileum and linear ulcers in the sigmoid colon. The patient had abdominal pain and a family history of inflammatory bowel disease. These findings led to the misdiagnosis of Crohn’s disease. This misdiagnosis persisted until the initiation of a biologic agent, exposing the patient to unnecessary and risky treatments with high costs. Although the completely normal findings on follow-up ileocolonoscopy performed after correct treatment could not be supported by the biopsies obtained at that time, we believe that the ulcers seen during colonoscopy may be explained by the local effect of the membrane surrounding the intestines on blood flow, leading to ischemia in some areas. Therefore, it is extremely important to evaluate the patient’s clinical, radiological, and laboratory findings together before making a misdiagnosis, which might have a lifelong negative impact on the patient. Awareness of characteristic ultrasonographic and CT findings may facilitate early recognition of abdominal cocoon in patients with chronic recurrent abdominal pain and bowel transit disorders.

## Figures and Tables

**Figure 1.**
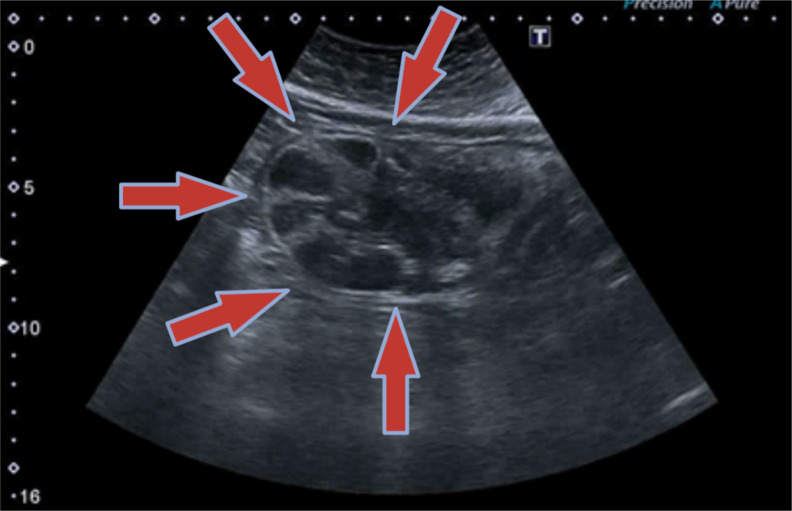
The membrane structure surrounding the small intestines in abdominal ultrasonography.

**Figure 2.**
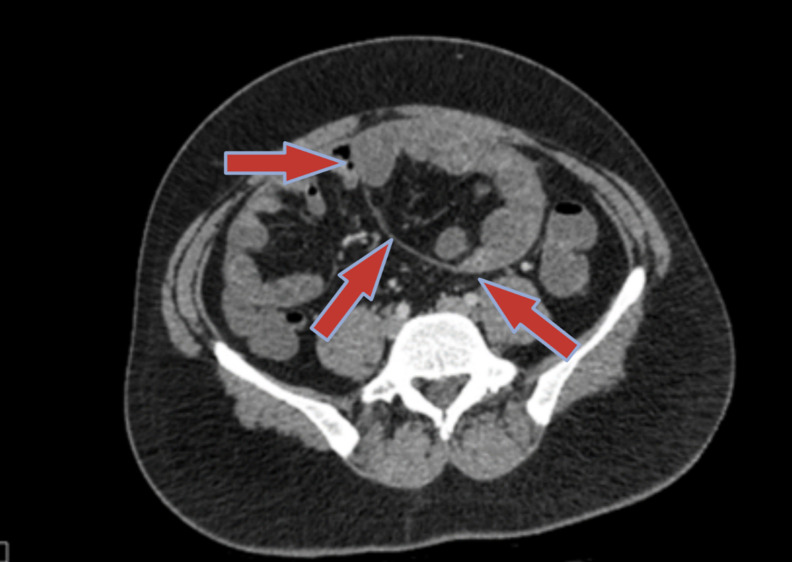
Computed tomography enterography shows edematous small intestines and fibrous tissue surrounding them, similar to those detected in ultrasonography.

**Figure 3.**
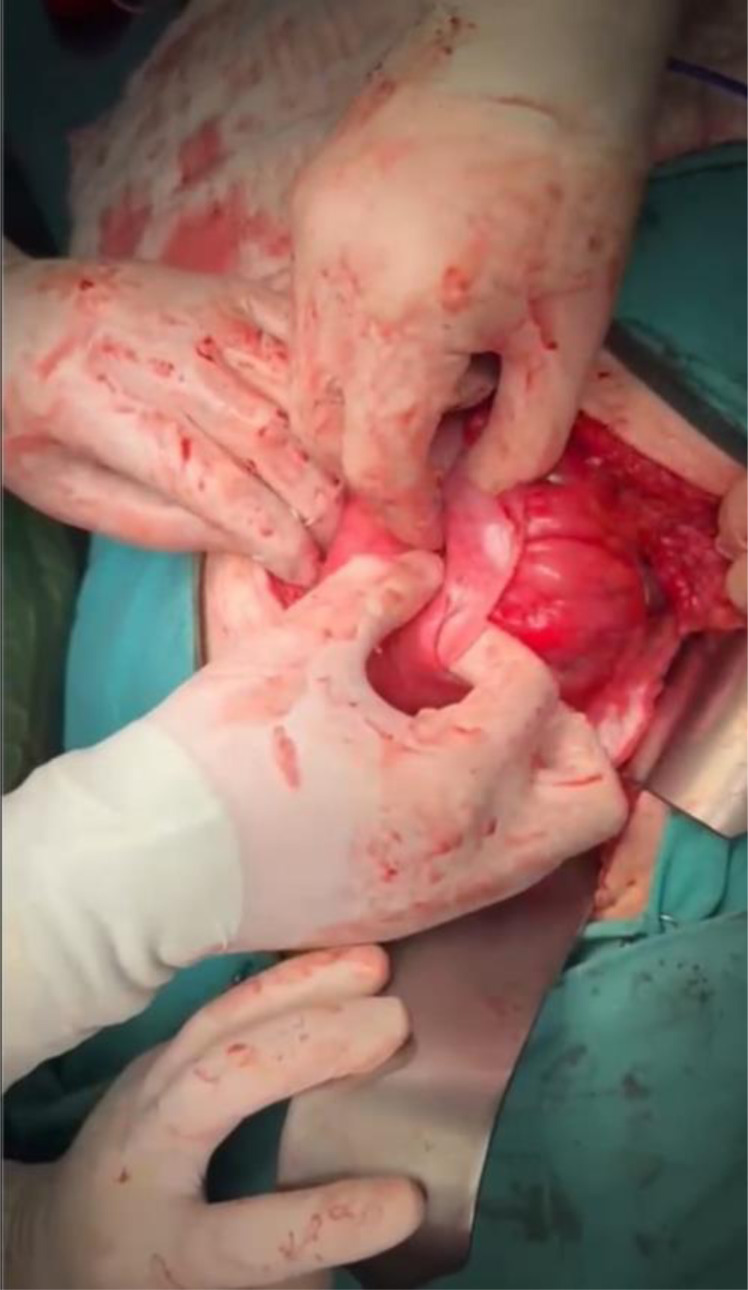
Fibrous membrane that surrounds the small intestine in laparotomy.

## Data Availability

The data that support the findings of this study are available on request from the corresponding author.
